# How to develop a sustainable telemedicine service? A Pediatric Telecardiology Service 20 years on - an exploratory study

**DOI:** 10.1186/s12913-019-4511-5

**Published:** 2019-09-23

**Authors:** Mélanie Raimundo Maia, Eduardo Castela, António Pires, Luís Velez Lapão

**Affiliations:** 1 Global Health and Tropical Medicine, GHTM, Instituto de Higiene e Medicina Tropical, IHMT, Universidade Nova de Lisboa, UNL, Rua da Junqueira 100, 1349-008 Lisboa, Portugal; 2 Pediatric Hospital, Centro Hospitalar e Universitário de Coimbra, CHUC, 3000-075 Coimbra, Portugal

**Keywords:** Case-study, Implementation research, Process evaluation, Telemedicine service, Telecardiology, Pediatric care, Sustainability, Universal access, Global health

## Abstract

**Background:**

Telemedicine services are promoting more access to healthcare. Portugal was an early adopter of telemedicine to overcome both its geological barriers and the shortage of healthcare professionals. The Pediatric Cardiology Service (PCS) at Coimbra University Hospital Centre (CHUC) has been using telemedicine to increase access and coverage since 1998. Their Pediatric Telecardiology Service has been daily connecting CHUC with 13 other Portuguese national hospitals, and regularly connecting with Portuguese-speaking African countries, through a teleconsultation platform.

**Methods:**

This study aims at exploring the Pediatric Telecardiology Service’s evolution, through a comprehensive assessment of the PCS’s development, evolution and impact in public health, to better understand the critical factors for implementation and sustainability of telemedicine, in the context of healthcare services digitalization. A case study was performed, with cost-benefit, critical factors and organizational culture assessment. Finally, the Kingdon’s framework helped to understand the implementation and scale-up process and the role of policy-making.

**Results:**

With the total of 32,685 out-patient teleconsultations, growing steadily from 1998 to 2016, the Pediatric Telecardiology Service has reached national and international recognition, being a pioneer and an active promotor of telemedicine. This telemedicine service has saved significant resources, about 1.1 million euros for the health system (e.g. in administrative and logistic costs) and approximately 419 euros per patient (considering an average of 1777 patients per year).

PCS presents a dominant “Clan” culture. The Momentum’s critical factors for telemedicine service implementation enabled us to understand how barriers were overcome (e.g. political forces). Willingness, perseverance and teamwork, allied with partnership with key stakeholders, were the foundation for professionals’ engagement and service networking development. Its positive results, new regulations and the increasing support from the hospital board, set up a window of opportunity to establish a sustainable telemedicine service.

**Conclusion:**

The Pediatric Telecardiology Service enables real-time communication and the sharing of clinical information, overcoming many barriers (from geographical ones to shortage of healthcare professionals), improving access to specialized care both in Portugal and Africa.

Motivation and teamwork, and perseverance, were key for the Pediatric Telecardiology Service to tackle the window of opportunity which created conditions for sustainability.

## Background

Telemedicine services represent an opportunity to improve Universal Health Coverage (UHC) [1], often contributing to mitigate global health challenges like patient’s evacuations [2]. Telemedicine is well accepted as complementary healthcare services in Portugal, overcoming many barriers of the national system, and compensating for existing asymmetries and the lack of resources [1].

After 20 years, the telemedicine service of the Pediatric Cardiology Service (PCS) at Coimbra University Hospital Centre (CHUC) has enabled better coverage of the population within the central region of Portugal. A regular PCS’s day encompasses several different opportunities where clinicians apply telemedicine: going from clinical observations at emergency level, to in-patient and out-patient teleconsultations for first diagnostic, observation or follow-up, referring to surgical interventions or treatment. PCS also cooperates with most Portuguese-speaking African countries (PALOP). The PCS’s Telecardiology Service is probably one of the oldest telemedicine service running in the world.

Within the scope of Portuguese national healthcare services (NHS) new strategy, telemedicine has been identified as one of the axes targeting quality improvement and effectiveness in primary healthcare [3]. The motivation is to provide person-centered services supported by a clinical governance culture. Portugal can be considered as an early adopter of telemedicine, while looking for overcoming its geological barriers and shortage of professionals [4]. Moreover, telemedicine services have been addressing patients evacuations to Portugal from countries belonging to the Community of Portuguese-speaking countries (CPLP) [5].

The CPLP’s Ministries of Health have recently expressed their commitment for the promotion of Information and Communication Technologies (ICT) targeting Universal Health Coverage (UHC) at a sustainable cost [2]. Portugal (e.g. PCS telemedicine service), Brazil (e.g. RUTE health teleeducation network) and Cape Verde’s use of telemedicine to address evacuations (e.g. National Telehealth Centre [6]) are the most notable cases.

The Portuguese NHS, like many other health systems, is facing a shortage of general practitioners^1^ (GP) and specialist physicians, such as Pediatric cardiologists [5]. NHS services’ coverage is often aggravated due to the fact that most specialty services are kept in central hospitals, which are located along the country’s coastline and distant from rural areas. Families living outside urban areas, have to deal with the problem of having to travel to attend consultations with specialists (e.g. for medical appointment, exam or treatment). Their travelling to central hospitals implies several additional costs, often putting the diagnostic confirmation and the follow-up at risk. The same scenario prevails in an emergency case. Emergency situations can often be tragic ones, with human lives loss. The lack of adequate transportation and the availability of services to stabilize the patient, or even of proper evacuation to the nearest hospital are quite frequent.

Specialized healthcare services are nonexistent in some regions, and with serious lack of human resources in most of the country’s interior.

ICT systems, when well-integrated within the clinical context, can improve access to quality professional education and training, as well as access to healthcare services [7]. ICT enables a forward-looking planning approach, in a long-term perspective, addressing real needs of health systems and their human resources, moreover regarding pediatric cardiology [8]. Telemedicine ensures the delivery of healthcare services, where distance can be critical [9], and the decision-support (e.g. evacuations [10]) efficiently enhancing global health system management and ensuring that services issues are fully addressed. Telemedicine services can play an important role in the healthcare services’ governance [11].

Telemedicine is often described as a sustainable service, demonstrating its important role in healthcare systems. Telemedicine services can also help address several healthcare barriers, increasing access to care [12], supporting emergency (e.g. cardiology for children and newborns, and fetal [13]), and avoiding unnecessary evacuations [6, 13], resulting in important cost-savings [14].

Nevertheless, formal evidence supporting the implementation process and effectiveness of telemedicine services is still limited, especially on pediatric care.

This study aims at comprehensively assessing the PCS’s development, evolution and impact in healthcare, to better understand the critical factors for implementation and sustainability of telemedicine, in the context of healthcare services digitalization.

## Methods

**Fig. 1:**
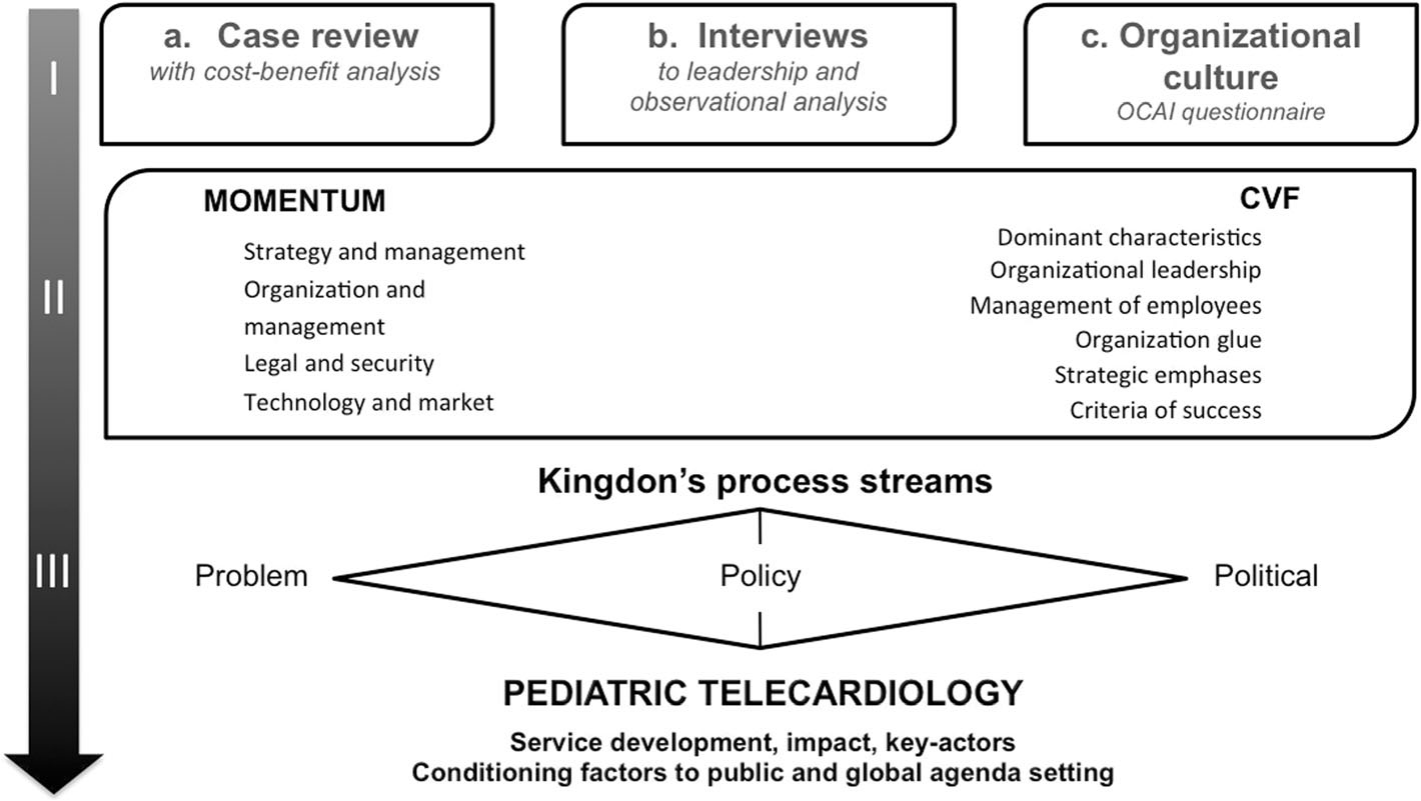
The case-study framework

This study follows an exploratory approach, observing the required deepness and robustness [15]. It was developed under the following methodology^2^:

I. A case-study was built using three sets of data: a case-review, interviews with PCS coordination and management, and finally, a questionnaire applied to PCS’s team using the Organizational Culture Assessment Instrument survey (OCAI) [16, 17] and some demographic questions.
II. All collected data were analyzed according to two complementary frameworks (Fig. 1) the **Momentum’s 18 critical criteria** [18] to launch telemedicine services and the **Competing Values Framework** (CVF) [17, 19], for organizational culture assessment.
III. The **Kingdon’s “three process streams” framework** [20], to better understand the process of telemedicine service implementation and development, identifying main actors and relevant factors to reach the policy agenda and eventually lead to a global reach [21].

### Study design and data collection

#### Case-review

Institutional data (e.g. on-site observations, provided official data and reports) and regulatory instruments (e.g. telemedicine laws and standards) were collected to perform the case-review. Physicians were observed in their different functions, especially during a set of scheduled teleconsultations (TC). The interaction between team members and real-life procedures were relevant to understand how the organization works. A S-shape logistic growth curve^3^ that explains the telemedicine technology adoption [22, 23], was fitted with the evolution data, tracing the TC trend, from 1998 to 2016.

A cost-benefit analysis assessed both the cost impact for (1) the population (Eqs. 1.a and 1.b) and for (2) the healthcare system (Eqs. 2.a, 2.b and 2.c), to better inform decision-makers [24]. The cost analysis was based on the direct and indirect costs comparison, from 2007 to 2014. It was taken into consideration what the costs would be, with and without the intervention (the telemedicine service implementation), as well as the benefits for the patients, families, organizations and health system [25].

(1) For this calculations, it was estimated an average of two TC per patient. The average travel cost for these two TC per patient can be calculated by simulating the round trip each patient would have to do to attend the pediatric or fetal cardiology appointments.

Average estimations were calculated considering several possible alternatives: (a) by ViaMichelin route planner [26], including petrol cost, with or without toll fees; (b) the cost of 0.36 euros per Kilometer, established by the national law for own vehicle transportation (value paid by public organizations); (c) considering transportation by ambulance, as established by the national law [27, 28]. The calculation formulas for estimating the total minimum and maximum costs for the patients are represented as:

Eq. 1.a $$ {\mathrm{C}}_{min}=\varSigma \left(\left({\mathit{\operatorname{Min}}}_{Hi}\ \left({T}_A\right)\times {TotalTC}_{Hi}\right)\right) $$

Eq. 1.b $$ {\mathrm{C}}_{max}=\varSigma \left(\left({\mathit{\operatorname{Max}}}_{Hi}\ \left({T}_A\right)\times {TotalTC}_{Hi}\right)\right) $$

where:

- *C*_*min*_: Total minimum estimated cost (Euros), considering all the performed Teleconsultation, *TC*.
- *C*_*max*_: Total maximum estimated cost (Euros), considering all the performed Teleconsultation, *TC*.
- *Hi*: Refers to each partner Hospital (site B; e.g. Aveiro, Castelo Branco, Covilhã).
- *T*_*A*_: Travel cost A, estimated for each *Hi*, considering 2 performed *TC* per patient. It is the minimum possible cost for travelling from *Hi* to CHUC, per patient, for 2 *TC*, considering the three alternatives: (a) An average estimation calculated using ViaMichelin route planner, including petrol and toll fees; (b) An estimation using the cost of 0.36 Euro/Kilometer (established by a national law applied in Public organizations); (c) In case of emergency, using an ambulance on request, which considers the cost of 3 Euro/50 Kilometer plus 0.15 Euros for each additional Kilometer, according to the national law.
- *TotalTC*_*Hi*_: Total *TC*, performed by each *Hi.*

(2) Telemedicine cost-savings for healthcare system were estimated considering the difference between the cost of an estimated face-to-face consultation cost and one TC. Administrative and logistic cost was estimated using referenced standard prices for an out-patient consultation (i.e. according to a national legal norm from 2016). This law establishes that, for each teleconsultation provided, the PCS would be paid the same amount corresponding to one out-patient consultation at the hospital of origin (site B). The calculation formula for estimating administrative and logistic cost-saving through telemedicine is represented as:

Eq. 2.a $$ {\mathrm{C}}_{withTC}={C}_{withoutTC}-{C}_{admLog} $$

where:

Eq. 2.b $$ {C}_{admLog}={TotalTC}_{Hi}\times {StPrice}_{Hi} $$

Eq. 2.c $$ {C}_{withoutTC}=\left({TotalTC}_{Hi}\times \left({StPrice}_{Hi}+{StPrice}_{CHUC}\right)\right) $$

considering:

- *C*_*withTC*_: Estimated administrative and logistic cost-saving through telemedicine (Euros); it is calculated using the estimated administrative and logistic total cost without telemedicine (*C*_*withoutTC*_) and the estimated administrative and logistic real cost (*C*_*admLog*_).
- *C*_*adminLog*_: Estimated administrative and logistic real cost (Euros) for the total performed *TC* by each hospital Hi; it consider the out-patient consultation standard price *(StPrice*_*Hi*_*) established by national law for each hospital, Hi.*
- *C*_*withoutTC*_: Estimated administrative and logistic total cost without telemedicine (Euros) for the Total performed *TC* by each hospital Hi; it consider both the out-patient consultation standard prices *(StPrice*_*Hi*_ *and StPrice*_*CHUC*_*) for the partner Hospital (Hi; site B) and the CHUC (site A).*
- *Hi*: Refers to each partner Hospital (site B; e.g. Aveiro, Castelo Branco, Covilhã).
- *TotalTC*_*Hi*_: Total *TC* (Euros*)*, performed by each *Hi.*

#### Interviews with key-members of the organization

The selected organization’s key-members were both the PCS’s former and current director. The ICT director was also included due to the technology nature of the telemedicine service. The three have been in the organization since the inception of the telemedicine service. During the field study, a 30-min semi-structured interview was conducted with each key-member. This step was important to study the leadership’s perception and expectations for further developments. The interviews were also important to identify the different stakeholders in the telemedicine agenda setting, and their roles in it.

#### The organizational culture assessment instrument survey (OCAI)

A questionnaire OCAI [16], was answered by several members of the PCS team, as the first part of the CVF framework [17, 19], enabling the organizational culture assessment and the CVF analysis. It was returned by 15 PCS team-members, aged 26–66 years-old (median: 37.0; Interquartile range, IQR: 32.5–49.0), 60% female. The questionnaire was completed by all team members during the service weekly meeting. They were mainly pediatric cardiologists with different functions and responsibilities, diagnostic and therapeutic technicians and an administrative assistant. It took each about 10 min to answer the questionnaire.

The survey data was analyzed, as proposed by the CVF authors, by calculating an average score for each A, B, C and D alternatives, and the “Now” and “Preferred” dimensions [19]. Each of these two scores is related to a specific organizational culture typology. Comparing “Now” to “Preferred” perspectives, one can estimate the predisposition towards future organizational change [29]. The OCAI tool was recently validated for the healthcare context in Portugal [17].

### Critical factors’ identification and organizational culture assessment

**Fig. 2:**
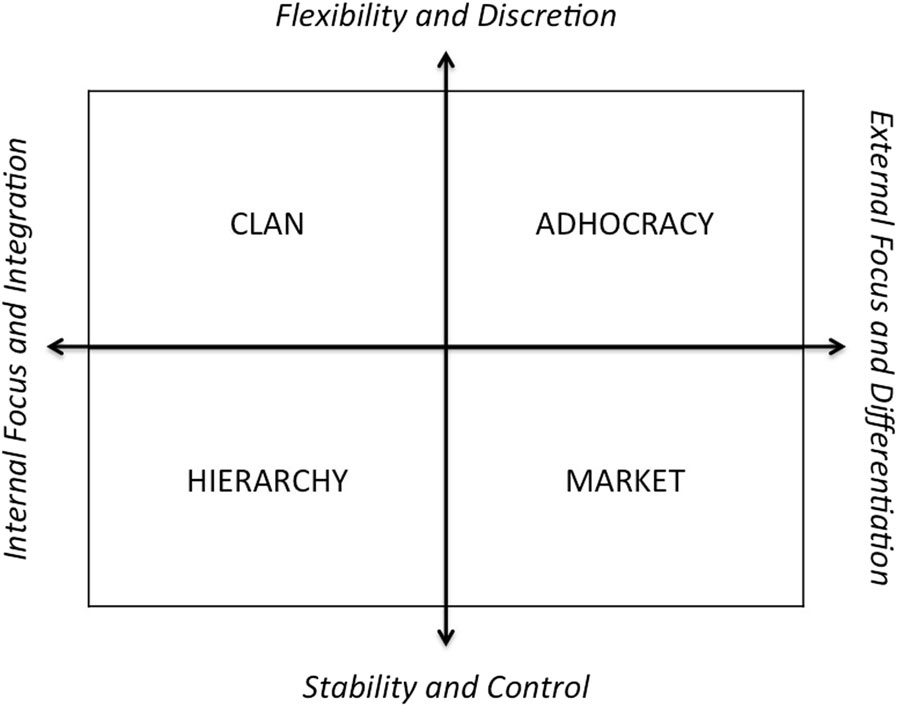
The Competing Values Framework, CVF [19]

The critical factors for a successful implementation of telemedicine and the organizational culture characteristics were identified. The following two steps helped to consolidate the case-study:

- Analyzing the Momentum’s 18 critical success factors for deployment [18], out of the collected evidence (i.e., from documents, questionnaires and the performed interviews), the situation points to the potential future challenges and possible directions for further development. The telemedicine service’s sustainability and scale-up were studied considering multiple criteria [18, 30, 31]: strategy, organization, management; legality, safety issues; technology innovation and constraints; market evolution and demand.
- The OCAI [17, 19] considered PCS workforce’s perceptions and preferences regarding telecardiology service’s evolution. It helped identifying the characteristics of the team’s culture. The CVF model (Fig. 2) analysis enables both the organizational process and strategy assessment, taking also into account future evolution expectations [17]. It introduces four major culture types - “Clan”, “Adhocracy”, “Market” and “Hierarchy” - based on Mintzberg’s organizational typologies [32].

### The Kingdon’s process streams

To study the role of policy in developing the telecardiology service, the Kingdon’s windows of opportunity framework [20] was applied. This methodology was previously validated for the Portuguese healthcare context [21].

Stakeholders, governmental and non-governmental ones, when organized and facing a “window of opportunity”, can streamline and transform public processes and their acceptance, leading to new policies. The full compliance with the three streams is absolutely necessary to a subject to stand out from the policy agenda and really make impact.

The “problem stream” leads to the recognition of the problem and brings it up into the public agenda. The “policy stream” generates validated alternatives for policy makers. After that, the “political stream” is the process, by which political actors accept and take a policy into their agendas and start advocating it towards its opponents.

Each stream addresses actual necessities and future visions but also considers national and international trends and should be aligned with the political agendas. The right timing to launch an innovation regulates the gathering of support, by reaching the potential stakeholders, and establish partnerships. Different forces from civil society, professional community, among others, are crucial to converge the process to a successful public intervention, with sustainability. This analysis will enlighten us about how the team’s decisions adjust to each of the streams, determining and redirecting its development path, overcoming barriers.

This empirical observational study presents some limitations, mostly inherent to the professional context. The study operationalization was complex, since we are dealing with different data sources: PCS registers, data until 2006; SONHO^4^ database, after 2007; institutional reports; individual experiences and testimonies. Due to the limited availability of recent data (from 2014 onwards), a more detailed analysis was only possible, from 2007 to 2014.

Participation was voluntary but subject to possible self-selection biases. The team demography was heterogeneous, both in cultural background and technologies acceptance level. However, as the aim was to study the team, this was only possible if we included everyone, and by considering both levels, the individual answers and group dynamics.

The study purposely selected telemedicine service key-members to understand how they acted as “champions” while pushing for change during the last 20 years.

## Results

A comprehensive methodological framework was set at three levels: (I) the case-study review, based on data collection, (II) the telemedicine service and organizational culture assessment based on combining the Momentum and the Competing Values (CVF) frameworks, and finally (III) the Kingdon’s framework, for better understanding the windows of opportunity determinants for the telemedicine evolution.

### Case-review

#### The telemedicine service’s characterization

Since 1977, the then designated Pediatric Cardiology Unit, has been a central element in delivering differentiated and specialized healthcare to children and pregnant women. Soon they turned-out to be the reference service, covering the central region of Portugal, giving support to Coimbra’s Pediatric Hospital (CPH), the University Hospitals and both Bissaya Barreto and Daniel de Matos Maternity Hospitals. In 2011, they became part of the CHUC’s Pediatric and Fetal Cardiology service, reorganized to fit the new pediatric hospital’s facilities. The move for the new facilities, specially dimensioned for its purpose^5^, was the opportunity to expand the service range to younger people, up to 18 years-old, counting on a new and more structured technical and human resources’ organization [25].

Telemedicine’s original idea occurred during a visit to Mayo Clinic in the USA in 1995. This experience motivated the idea of using telemedicine to improve healthcare coverage in Portugal [5]. Later, Dr. Eduardo Castela (the PCS service’s Director; and a Mayo Clinic trainee), shared his experience with Lusitânia Fonseca, Portugal Telecom Engineer and Head of the Innovation Department. She immediately saw an opportunity there, and in 1998 the first telemedicine consultation was a reality.

Bilhota Xavier (Pediatrician at Leiria’s Santo André Hospital, about 73 Kilometers away from Coimbra) was the partner needed to build-up a successful telemedicine pilot between Coimbra and Leiria. Both Castela and Xavier believed on the benefits of creating an ICT-based service and that the scale-up to various hospitals would help to mitigate the access problem [5].

The first telemedicine transmission was successfully accomplished between the CPH and both Júlio Dinis Maternity (in Porto) and Santo André (in Leiria) hospitals, on October 14th, 1998. The team’s concern about documenting, studying and sharing their own experience lead to a close collaboration with the academy [4].

**Fig. 3:**
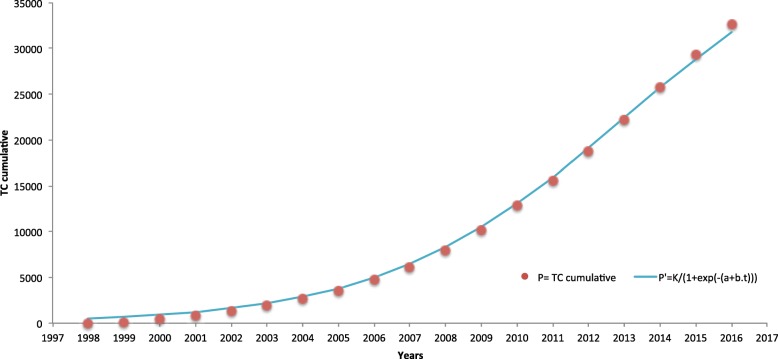
Telemedicine service logistic growth curve. Telemedicine service adoption for out-patient consultations, 1998–2016. P: cumulative performed teleconsultations; P′: logistic fitted curve (*r* = 0.9997), calculated with the Griliches’ model [22]

The PCS performed, from 1998 to 2016, a total of 32,685 out-patient TC, with a pattern that follows an S-shape logistic growth curve (Fig. 3). Well-fitted to the data (correlation coefficient, r = 0.9997), the logistic curve shows the service’s gradual (although rather slow) diffusion [23]. If the TC progression maintains this pace, its maximum grow, stated by the S-curve ceiling (estimated *k* of 44,250 TC), is only expected to happen in the future (about 2050).

**Fig. 4:**
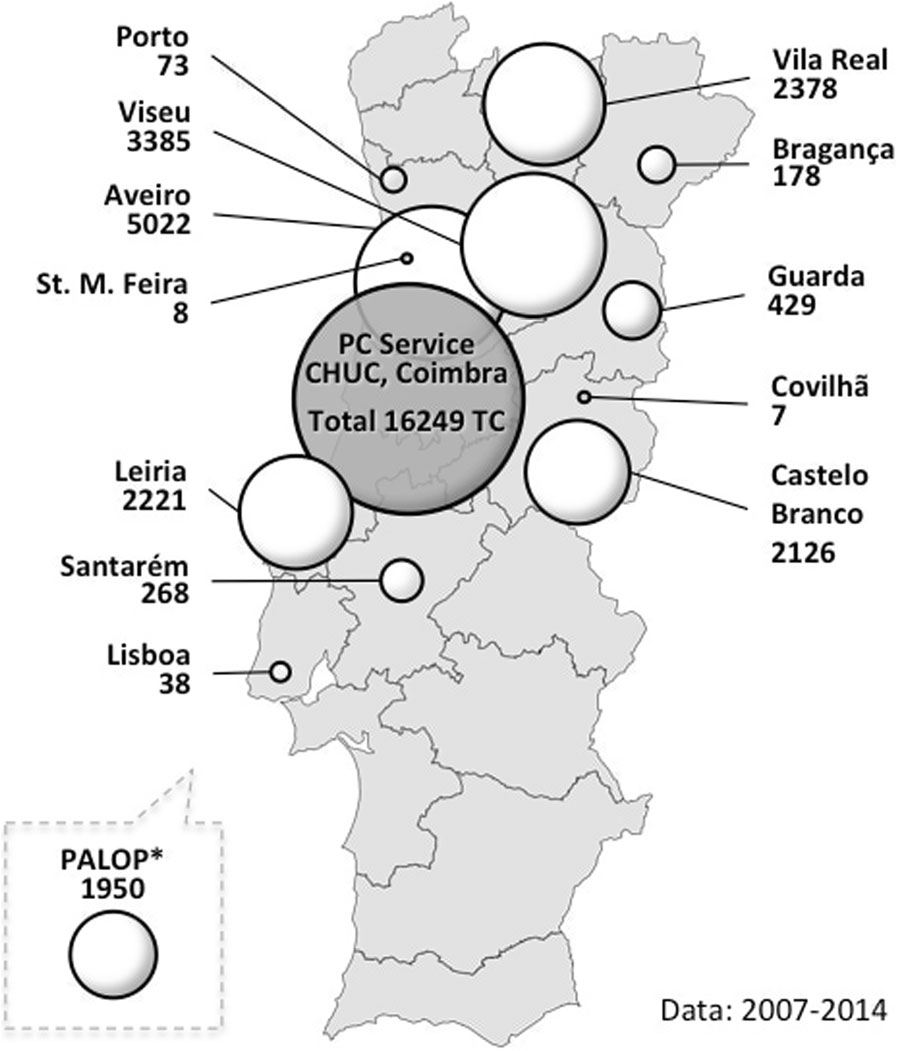
Teleconsultations (TC) distribution. Schematic representation of the PCS’s activity and its geographical distribution (* PALOP total includes data from 2007 to 2016. For the rest, data from the last 2 years are not officially known yet). Source: original

The PCS successfully connected CHUC with several Portuguese NHS’s district hospitals (currently 13), and most of the PALOP’s main hospitals, through the TC platform (Fig. 4), encompassing both pediatric and fetal cardiology and seldom tropical diseases complications (Table 1).

**Table 1:** Evolution of telemedicine, considering only out-patient consultations, provided by the PCS, 2007 to 2016

	Most representative Telemedicine Sub-specialties (for NHS)		Teleconsultations (for NHS)			Pediatric Cardiology out-patient consultations			Teleconsultations by Pediatric Cardiology out-patient consultations		
	Telecardiology (total of 13,903 patients)	Fetal Telecardiology (total of 319 patients)	Total	First Consultation	Subsequent Consultations	Total	First Consultation	Subsequent Consultations	Total	First Consultation	Subsequent Consultations
2007	1363	0	1363	1288	75	6734	2783	3951	20.2%	46.3%	1.9%
2008	1842	36	1878	1872	6	7662	3680	3982	24.5%	50.9%	0.2%
2009	2137	60	2197	2193	4	8841	4212	4629	24.9%	52.1%	0.1%
2010	2613	89	2702	1938	764	9979	4020	5959	27.1%	48.2%	12.8%
2011	2579	124	2703	1780	923	10,235	3892	6343	26.4%	45.7%	14.6%
2012	3094	78	3172	1947	1225	11,174	4405	6769	28.4%	44.2%	18.1%
2013	3462	0	3462	2006	1456	12,229	4693	7536	28.3%	42.7%	19.3%
2014	3510	0	3510	2055	1455	12,142	4561	7581	28.9%	45.1%	19.2%
2015	3394	214	3608	*n.a.*	*n.a.*	*n.a.*	*n.a.*	*n.a.*	*n.a.*	*n.a.*	*n.a.*
2016	3131	218	3349	*n.a.*	*n.a.*	*n.a.*	*n.a.*	*n.a.*	*n.a.*	*n.a.*	*n.a.*
Total	27,125	819	27,944	15,079	5908	78,996	32,246	46,750	35.4%	46.8%	12.6%

*n.a* non available or non applicable. Source: PC Service’s register and National database (SONHO, 2015, 2017)

From 2007 to 2016, a total of 1647 TC were performed with Angola (linking to both Luanda and Benguela hospitals), 477 TC with Cape Verde (linking to both Praia and Mindelo hospitals) and 82 TC with São Tomé and Príncipe’s central hospital. The PCS continues to perform telemedicine on a weekly base. Soon, it is expected to include Guinea-Bissau in the network.

Over the last decade, TC were responsible for about 35.4% of the total out-patients, provided by PCS (Table 1), with a predominance of first consultations (46.8% of the total first consultations). The 12.6% of subsequent consultations, for patients’ follow-up and monitoring, shows the telemedicine service consolidation, which is critical indicator for specialized hospital care access.

**Fig. 5:**
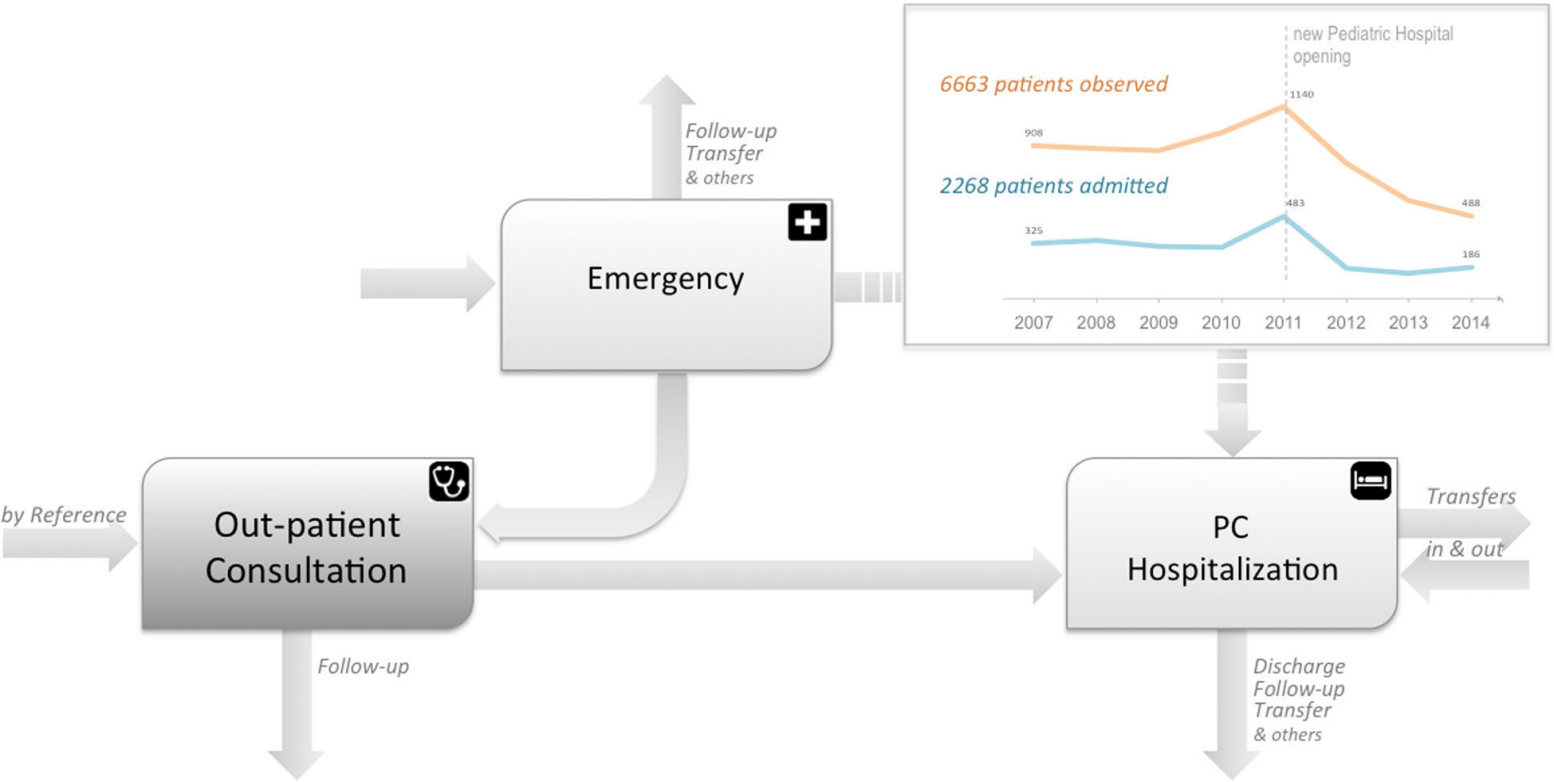
PCS’s patients flow. PCS’s range of action and patients flow, in CHUC. Out-patient consultation: singular act of observation and procedure. PCS hospitalization: admissions by the PCS, with hospitalization of more than 24 h. Emergency: Emergency Room procedures and observations

Telemedicine activity focuses mostly on out-patients. However, the complexity of PCS’ patients-flow shows different patterns. PCS provide as well emergency consultations for the hospital’s emergency service (Fig. 5).

In 2011, the new CPH facilities, originated a peak of admissions and observations, at emergency level, performed by the PCS team. Both the PCS reorganization and the fact that they don’t have dedicated beds allocated for in-patients, they need to rely on the Pediatric Medical Service capacity (located in the same building), contributes to a more efficient management. TC helped to reduce both emergency admissions and emergency consultations to almost half their initial values. The majority of patients’ hospitalization (75%; given by discharged patients numbers) are previously scheduled [25].

#### Cost-benefit analysis

**Fig. 6:**
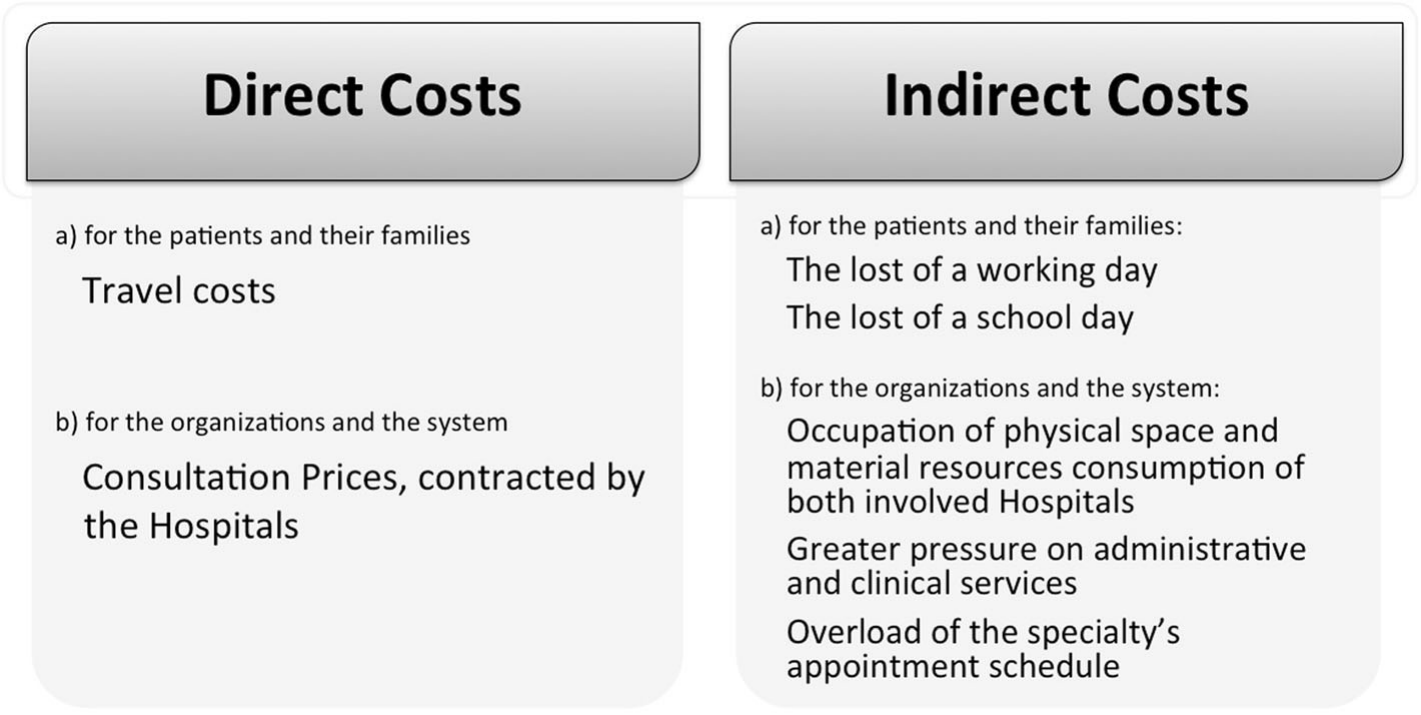
Direct and indirect costs to consider in telemedicine impact assessment. Main costs to consider, mostly related to organizational and geographical constraints

A positive cost-benefit balance is critical for telemedicine service sustainability [33]. Telemedicine service costs and benefits were estimated, regarding the health system, patient and families (see Additional file 1 for more detail). Both the patients (and families) and PCS’s perspectives were used. To understand the impact of telemedicine, we considered direct costs and indirect costs, often the hidden part of the “iceberg” (Fig. 6).

The PCS patients, including out-patient (97.7% of total TC) and emergency (2.3%) TC, are mainly from the center and north regions, especially from Vila Real, Viseu, Aveiro, Castelo Branco and Leiria cities (206, 92, 60, 150, 73 Kilometers away from CPH, respectively; Fig. 4). They present an increasing trend of TC (see Additional file 1). Given the distance between the hospitals and CHUC, the costs calculation considered direct costs for patients and families (e.g. the family member taking the children to the consultation), and organizations and the system. It was estimated travel costs and consultations fees (Eqs. 1.a and 1.b; see Additional file 1). Avoidable travel costs may reach 419 euros, maximum per patient, considering the average of 2 TC per patient (i.e. a first consultation and a follow-up). The total avoidable travel costs, per round trip^6^ was estimated from 337 to 1265 thousand euros, considering the minimum and maximum possible distances within the region, respectively. Family time spent estimates are indicative of at least one full day loss in their routines (missing school and work), an important indirect cost to be considered.

The cost reduction for the organizations and for the system included the estimated administrative and logistic cost-saving through telemedicine (Eqs. 2.a, 2.b and 2.c). Telemedicine has saved approximately 1.1 million euros for the health system, the difference between estimated face-to-face consultation cost and TC cost (see Additional file 2). By performing TC, the administrative and logistic costs reduce to half. With telemedicine, the national health system only have to reimburse the rate (or out-patient consultations standard-price, [34]) corresponding to the patient’s first point-of-contact (the hospital from site B, that refers the patient to site A, the CHUC). Moreover, TC decreases pressure in administrative and clinical services, as well as in the appointment scheduling.

As a natural evolution of the innovation, after a slow growth period for “acceptance” [22] and implementation, corresponding to the “pilot period”, the practice started gaining significant supporters. In order to promote and regulate TC, by stimulating the adoption of hospitals, some important regulatory tools were launched by the Ministry of Health. By 2006, the telemedicine consultations were finally and officially recognized.

It was established a consultation fee (30 euros [35]), whereas both hospitals are allowed to register and account for it (in reality, a cost of 60 euros for the health system). Following measures updated the fee value (31 euros and 80.34 euros, respectively) [36, 37].

**Fig. 7:**
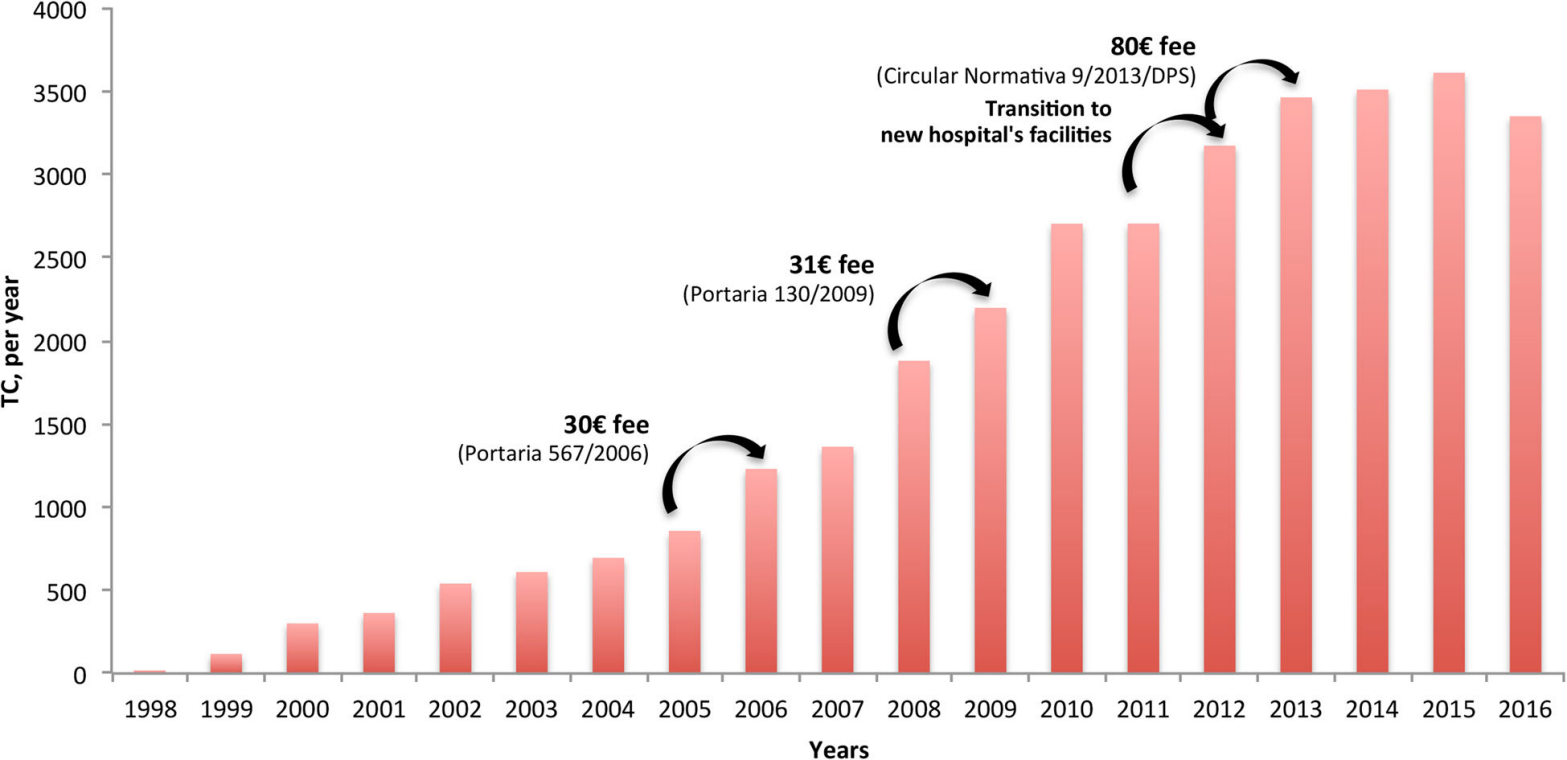
Performed TC evolution. Telemedicine evolution for out-patient consultations, 1998–2014. Arrows: main growth stimulating events

Such regulatory steps encouraged the increase of TC in the following years (Fig. 7). The reorganization and the new facilities of the PCS service latter in 2011, when the service moved to the new hospital, also permitted to increase TC capacity.

The slight observed decrease in TC, from 2015 to 2016, happened due to the technology change related with the platform upgrade, which involved a new procurement and commissioning process. Interviewees also suggest it could be related to a new tendency to practice informal telemedicine, by using digital applications (e.g. WhatsApp), which “could facilitate the process of sharing information”.

### Interviews with key-elements and observational analysis

#### Observational analysis - understanding the process

**Fig. 8:**
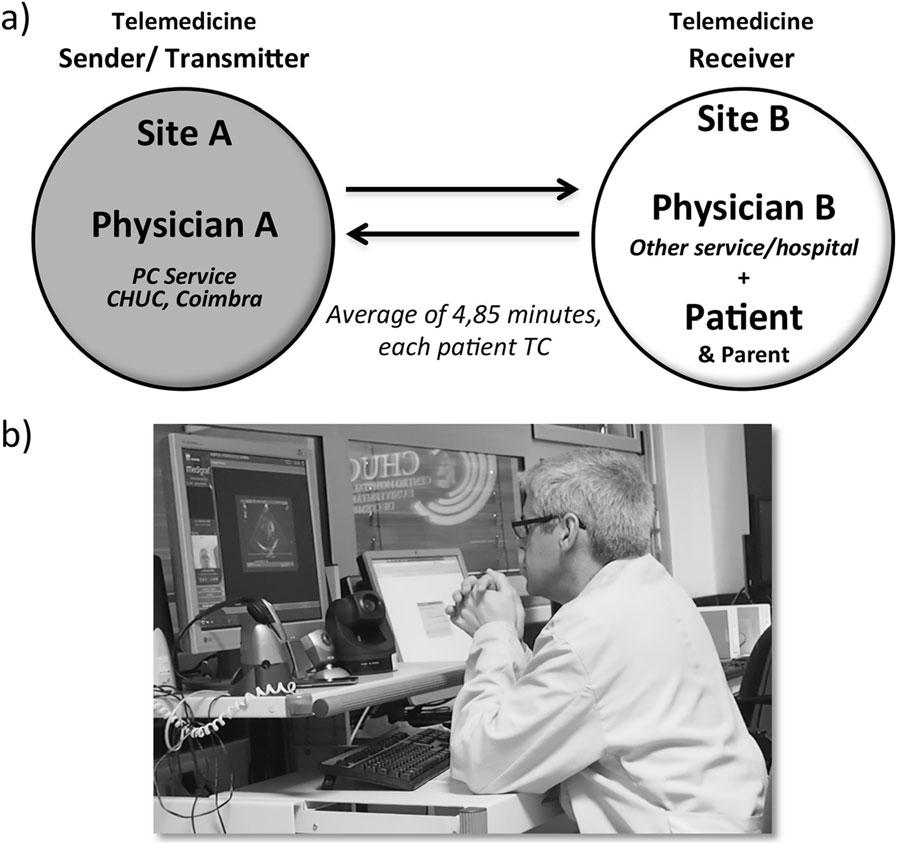
Schematic representation of a PCS’s teleconsultation. **a** TC’s communication flow between sites A (Sender) and B (Receiver). **b** Example of a telemedicine session; perspective from site A. Source: original

The telemedicine technological platform is a simple but sophisticated equipment. The Medigraf’s TC platform, was developed by Portugal Telecom with direct guidance from Dr. Castela [5]. It enables real-time visualization and the record of an echocardiogram, while oral communication and data files’ exchange occurs between the specialist physician, Physician A, and the other site’s physician, Physician B (Fig. 8). The medical data is recorded in the databases of the national health information system network (RIS). In addition to these information systems, the PCS’s activity relies also on complementary diagnosis procedures. The most frequent are echocardiography (Echo) - Doppler, transthoracic two-dimensional (12-lead), and electrocardiography (ECG) - Holter (until 24-h), stress test.

A telemedicine session observation, focusing on the physician A’s procedures and interaction with the equipment, provided the understanding on how telemedicine allows the optimization of the physician A’s schedule and the better management of his functions. The observed telemedicine session was transmitted from CHUC to another national district hospital, in a total of 45 min for 5 TC (9 min, in average; each TC took near 6 min, plus 52% of hold time; Table 2).

**Table 2:** Comparison between TC duration in both sites A (Sender/Transmitter) and B (receiver)

Average TC duration in site A, by Patient (minutes)	Estimated average duration of the TC in site B, by Patient (minutes)
5.82	8.82

**Fig. 9:**
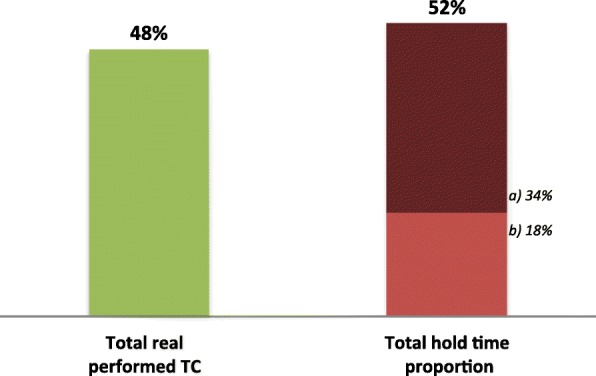
Telemedicine’s observed time distribution. a) Total extra hold time during patients TC, 34%. b) Total hold time between patients, 18%

From the PCS perspective, the TC schedule is managed by assigning each patient, considering the hospital of origin (site B) to a designated consultation day and physician (site A). This planning procedure eases management at both hospitals. The patients with appointment in B usually are previously prepared and aligned for the TC with A, for optimization. Preparation does not require an additional consultation. It is mostly scheduling a pediatric consultation where a nurse helps preparing the patient for a telecardiology exam that takes about 5 min). Despite those efforts, a major part of the observed time (52%) was considered as waste time, spent on hold (“hold time” in Fig. 9), with the patients’ turnover, and documents’ sending and receiving proceedings. “Hold time” varies from hospital to hospital, as some are more efficient than others regarding these proceedings.

#### Organizational culture assessment

**Fig. 10:**
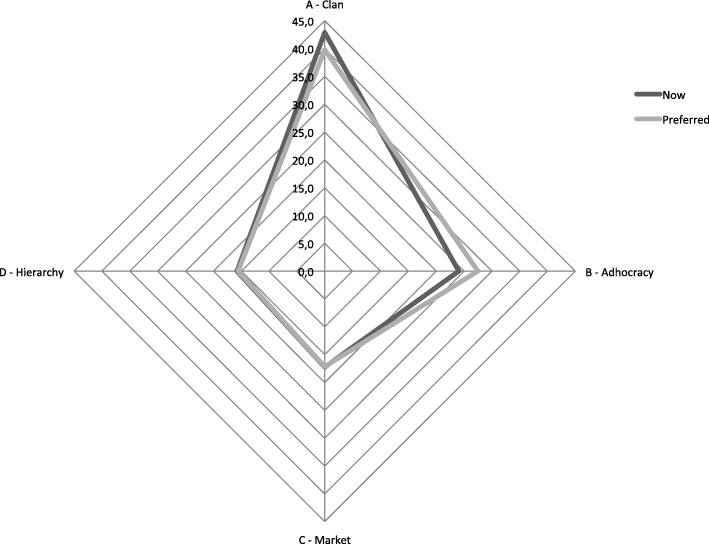
Culture profile, now and preferred for the next 5 years

The PCS organizational profile was analyzed using data from the questionnaire OCAI [17, 19], considering dominant characteristics, organizational leadership, employees’ management, organization glue, strategic emphases and criteria of success. According to the estimated OCAI’s scores (Table 3), the culture profile, now and preferred (considering a 5 years’ goal), is represented in the radar chart (Fig. 10). The identified dominant culture is of “Clan” type, with strong “Adhocracy” characteristics (second dominant). It is expected to give the necessary autonomy to better face the daily team challenges. Nevertheless, a mix of “Market” and “Hierarchy” values are still present in the organization. “Adhocracy” culture seems to be gaining ground in the organization (Fig. 10).

**Table 3:** OCAI’s scores for organization culture assessment

Culture alternatives	Now average score/categories	Preferred average score/categories	Differences (Preferred - Now)
A - Clan	42.9	39.8	−3.1
B - Adhocracy	24.1	27.5	3.4
C - Market	17.2	17.2	−0.1
D - Hierarchy	15.8	15.5	−0.2

### Critical factors identification and organizational culture assessment

#### The culture trend

In a more stratified analysis, it can be verified that all the six categories have the “Clan” culture as the dominant one. “Clan” is often the dominant culture in healthcare services [5]. The top score for the dominant characteristics (Table 4) indicates that people sees the service as a personal place, the team as a family and a strong sharing relation. “Organizational leadership” values for the “Now” moment points to a leadership that is such an example as tutor, facilitator and caring, followed with a perception of an entrepreneur and innovative leadership. This dimension suffers a change direction to the “Preferred” future, strongly valuing “Hierarchy” values, by having the vision of a leadership who develops itself into a control and efficiency direction. The “Strategic emphasis” dimension presents a very even values mix, once more being prevalent on the flexibility and discretion driver, based in the “Clan” and “Adhocracy” characteristics oriented to collaboration and creativity. This type of organizations emphasizes human development, facing new challenges and prospecting for opportunities, though. “Management of employees” and “Criteria of success” dimensions focus on teamwork, with a collaborative process in an integrated way. On the issue of the “Organizational glue”, loyalty and mutual trust, valuing commitment, are the dominant perception, although with emphasis on results achievement and goal accomplishment.

**Table 4:** Culture trend by characteristics categories

Categories	Now Average score/categories				Preferred Average score/categories				Culture Change by categories
	A	B	C	D	A	B	C	D	
1. Dominant characteristics	**47.0**	27.0	18.0	8.0	**37.5**	35.0	20.4	7.1	
2. Organizational leadership	**37.7**	28.3	15.3	18.7	28.9	23.9	9.3	**37.9**	
3. Management of employees	**50.0**	18.2	13.6	18.2	**45.0**	20.4	17.5	17.1	
4. Organization glue	**41.4**	17.9	30.0	10.7	**38.8**	25.4	29.6	6.2	
5. Strategic emphasis	**36.4**	35.0	13.6	15.0	33.5	**41.5**	14.2	10.8	
6. Criteria of success	**45.0**	18.0	13.0	24.0	**55.0**	18.8	11.9	14.2	
Total score	42.9	24.1	17.2	15.8	39.8	27.5	17.2	15.5	
				100.0				100.0	

Text in bold indicates the highest score for each category group

#### Critical factors for telemedicine deployment and sustainability

The Momentum’s Shamrock framework for telemedicine implementation, presents three main scopes (represented by each of the shamrock leaves): “People”, “Plan” and “Run” process (see Additional file 3). The shamrock stem lies in the context of the telemedicine service, where one should find both a cultural readiness and a compelling need consensus. The 18 critical success factors analysis required the collection of evidence from documents, questionnaires and interviews. The PCS was found to be in compliance with most the critical success factors, with strong strategy and organization, despite some vulnerability related to legal and security issues (e.g. most telemedicine service’s logistic and technical aspects were, until recently, not covered by national law).

### The Kingdon’s process streams

The “three-stream” analysis indicates different moments where policy agenda changed, generating windows of opportunity that created the conditions to TC service scale-up.

According to Kingdon’s theory [20], besides the governmental actors, action also engaged non-governmental groups of influence. On the one hand, we may have actors such as business, professional or practitioner and labor groups. On the other hand, one may find support or opposition from “public interest” groups. The balance between the parties contributes to the promotion of a new policy agenda, setting the new strategy for telemedicine services development, but remaining some differences between political agendas and interests. Technology came to be both positive and negative regarding the PCS development. It became a positive factor by enabling a successful care service, helping to overcome distance barriers. Nevertheless, it became the target for adverse blocking from the government, as a result of interest groups’ pressure. The initial problem was telemedicine implementation and recognition, which was fully addressed. The recent debate addresses policy issues like the use of standard technology to improve interoperability. For instance, the SPMS (entity of the Ministry of Health with procurement and supply chain management functions) pressured for the adoption of a different nation-wide telemedicine system (still lacking effectiveness) rather than Medigraf. This could result in negative pressure, instead of positive promotion. These interest groups’ action influence all the process, sometimes conditioning the course of government action. In July 2017, CHUC decided to upgrade the Medigraf system, allowing for faster and better services. This is a new positive factor for the PCS development.

## Discussion

### The PCS telemedicine service genesis

The PCS telemedicine service’s impact in Portugal’s central region public health services is clear, with more than 32 thousand performed TC.

Now, 20 years after, technology is still important, since communications’ providers and the technology itself can change but the service has to be guaranteed, not putting in risk the TC nor the safety of patients’ medical data. The PCS relies now a significant part of its workload on telemedicine, which is a demonstration that it is essential and relevant for its global performance.

Telemedicine services are an opportunity to connect Portuguese-speaking countries (CPLP) and promote better healthcare services [2]. The increasing numbers of telemedicine consultations, over the years, proves that this telemedicine service is central in giving more access to these populations. This is a good example that otherwise, without telemedicine, access would be more difficult or even impossible to distant patients. Based on strengths and opportunities, the PCS leverages itself by promoting privileged relations with other healthcare entities, and by improving its ability to follow-up technological innovations [38].

A strong organizational culture, proved to be capable of overcoming the difficulties, often associated with the process of growth [4, 5]. The PCS organization shows a dominant “Clan” culture, which reveals conditions for openness to innovation and creativity, based on the team work and leadership. According to Cameron and Quinn, when all of these characteristics are integrated in terms of quality management, the success rate increases significantly, benefiting people’s empowerment strategies, team building and open communication.

In a global context of telemedicine services for pediatric cardiology, similarly to other services in this field, the PCS telemedicine service is another example that results in an (1) increased access to healthcare by patients, in (2) improved HP quality of work, and also in (3) costs-saving for both patients and the health system.

The PCS is part of the CHUC’s Pediatric Hospital that provides a range of specialist pediatric services to local, national and international patients. Through the diagnosis or exclusion of congenital heart disease, whenever suspected by the pediatric departments of district hospitals, children without disease are excluded and those with disease are followed in partnership with district hospital medical staff [4]. The hospital provides world-class clinical care and training, pioneering new research and treatments in partnership with others for the benefit of children in Portugal and worldwide.

### Technology and human resources for health, combined to improve access

The success of a telemedicine service depends not only on the technological implementation process but on all process of promoting and managing it [39]. It was a slow but consistent process, as shown by the S-shape logistic growth curve (Fig. 3). The beginning of the telemedicine service presents a typical behavior of technology innovations. Griliches [22] stresses the idea that the setting of an “origin date” is complex and somewhat difficult to precise; since *“(...) it does not have a beginning*” [22]. We can, however distinguish an “availability” time period from an “acceptance” one. “Availability” is the direct result of first regulatory actions (Fig. 7). Regulatory measures had an important role in the scale-up of the service. Until “availability” conditions were established, HP had to deal with a period of slow “acceptance”. This resistance behavior could be related with the natural human resistance to an unknown technology but also to change in habits [40, 41]. Moreover, individual interaction to the system and the system’s design would influence it as well [42]. Although the technology solution (i.e., the telemedicine platform) was just giving its first steps, it was being constantly validated and improved by the users’ comments and suggestions.

Telemedicine service’s sustainability and effective scale-up require clear commitment to capacity building and improving digital literacy [2, 43]. Cultural readiness (e.g. trustable environment between physicians from different institutions, willingness for habits’ change, decision-making autonomy) and compelling need (e.g. stakeholders’ consensus on telemedicine advantages and on the problem that needs a solution such as the lack of resources) consensus are strongly associated to strategy and management activities. These Momentum’s drivers enable the context for transition from strategy, management and organization to operational levels (see Additional file 3).

The “People” critical success factor relates to leading people and their interaction with telemedicine service. Under this perspective, people was educated and user-friendly systems and equipment design were promoted. This is aligned to the organizational culture profile for this service. The same way, the “Plan” factor, which are related to previous setting up of all the necessary resources, were duly and previously ensured. A business plan was at the ground for action (see Additional file 3). Key-members mentioned the existence of a detailed business plan for action, developed by the PCS to cover rural area of central region (e.g. already considering travelling costs). Portugal Telecom, the key stakeholder responsible for the technology and telecommunications, developed their own business plan as well. Unfortunately, the research team didn’t had access to either, so it was not possible to deepen this analysis aspect.

“Run” factor is specially related to the legal and security issues, concerning to key-factors for underpinning the operationalization of a large-scale telemedicine service deployment. Technology and market issues are crucial for the running stage. In PCS, the “Run” factor was stronger at the beginning. In the early years, there was a lack of legal context for telemedicine service. However, that has recently changed. With intensive regulation efforts, the level of demand got bigger, though stimulating the telemedicine adoption [44]. More pressure came from outside, from government, as well as from academia and professional associations. Despite these efforts, there is the concern that the recent observed decrease of TC (from 2015 to 2016; Fig. 7) could be related to a new tendency to practice informal telemedicine by using social digital applications (e.g. WhatsApp). The argument seems to be that the apps simplify the sharing and communication process (e.g. in emergency cases). According to the interviewees, sometimes digital applications such as WhatsApp have been used to share files and impressions between physicians. This new trend has been recently documented in the healthcare context [45]. Confirming this issue in the follow-up, we may have to deal with new regulation, technological adjustment and educational actions, in order to keep complying with the Momentum’s critical factors (e.g. legal and security stream; see Additional file 3).

The reimbursement law, enabled that both services - the PCS (site A) and the client Hospital (site B) - received the payment for each consultation. Paying both sites represented a clear window of opportunity to push for telemedicine services.

Limited financing could constrain the possibilities of scaling-up. PCS mostly counts on public institutional funding. However, PCS has demonstrated strength enough to go further despite this limitation, by leveraging with team work and partnership network. This is why it could be a valid model that encourages other healthcare systems to work together with HP in rural or distant locations (e.g. CPLP context).

At implementation stage, PCS’s African partners had to deal with foreign investment for infrastructure (e.g. teleconference and diagnostic equipment; connectivity service). In these cases, teleconsultations are not accounted since they are covered by a protocol between foreign countries’ ministries of health and Portugal. The challenge in African countries has been somewhat similar to the Portuguese context, regarding the Medigraf’s update that implies an update and/or upgrade for other equipment (e.g. Echocardiography). Especially the African partners have been struggling with financing the upgrade of equipment to follow-up the service.

Concerning the future scale-up of the service, towards a global goal of widening the healthcare of pediatric and fetal telecardiology delivery, there should be considered the change or the maintenance of the ICT system. Other aspect is that the Medigraf system does not fully integrate with the RIS database. Since the operationalization of the technologies is converging to the integration of systems, we have to ask if that is a requisite for the future, or if it is dispensable, due to the risk of stressing the process without need.

### Service evolution and political window opportunity - is there space to scale-up?

The PCS’s path points out to motivation, teamwork and perseverance as key sustainability factors. The initial implementation of PCS telemedicine service, back in 1998, was accomplished due to the involved pioneer physicians’ willingness. However, most of the time, the telemedicine service was limited to the geographically closest partners, that caused a slower pace in spreading telemedicine. Only recently the government embraced this initiative, when telemedicine came to be a new stream for the health services delivery. When it began to receive more attention from policy makers, researchers, professionals and the users, several working groups were created for its study, development and dissemination. With telemedicine recognition [46], financing conditions became central. To address the development of a national telemedicine network new strategy [47], some other initiatives were tested (e.g., teledermatology screening) [44]. Telemedicine network only became available and duly regulated at national level, in late 2016, when the National Telehealth Centre was launched.

When health policy makers’ attention finally turned to create a national telemedicine strategy, one prominent consideration concerned to the budgetary relief this would provide to the country. The existence of multiple political forces contributed to define the telemedicine evolution, and the PCS as well. Pressure came indirectly either from business groups or practitioner groups that firstly ignored and delayed the PCS initiative. However, the physician’s willingness prevailed. PCS early proved TC’s accuracy for pediatric cardiology diagnosis [4]. Hence, PCS found increasing support from the Central Region Hospitals and physicians. Technology and the service quality became the positive constant and a PCS advantage.

## Conclusion

The PCS’s case shows that it is possible to design and implement a complex telemedicine service, in a sustainable way. But it takes time to reach maturity. Since 1998, the Pediatric Telecardiology Service has been successfully functioning in Central Portugal, connecting CHUC with district hospitals. This system also allows the team to cooperate internationally, with CPLP on a regular basis.

The “secret” of success identified was mainly a professional and motivated team, which had at their disposal a simple, but effective technology. Additionally, the creation of a network of partnerships with district hospitals and the active collaboration of ICT teams were paramount. Last, but not the least, the inclusion of TC reimbursements (and foreseen in the business plan) from NHS and the required time to improve and adapt to each client Hospital’s needs.

The PCS has now reached the national and international recognition, being a pioneer and an active promotor of telemedicine, globally. Both geographic and specialists’ coverage problems can be addressed with telemedicine solutions. Accordingly, the hospital resources should be more easily accessible and shared [48]. It represents an opportunity for a new political consensus.

This case confirms that a sustainable telemedicine service depends on motivated teamwork, leadership engagement and supporting regulations (e.g., to guarantee financial sustainability). The follow-up of PCS’s telemedicine service should be ensured, in order to keep monitoring and adjusting the telemedicine service to the context evolution. Future lines for research should be focused on keeping track of technological developments but mostly the new user trends. Informal telemedicine practicing (e.g. using digital apps) could be rising. If confirmed as a trend, we could be facing a new regulatory, technological and educational challenge.

## Supplementary information

**Additional file 1.** The cost impact of telemedicine for patients and their family, per municipality and totals. Represented through time spent, travelled distance and financial direct costs, calculated through three methods. Includes out-patient consultations and emergency acts, by telemedicine.

**Additional file 2.** The organizational cost impact of telemedicine, for the system, per municipality and totals. Given by out-patient consultations’ price (representative of the clinical and administrative activities related with each consultation act), contracted by each hospital, according to the respective national norm [34]. Includes out-patient consultations and emergency acts, by telemedicine.

**Additional file 3.** The Momentum’s 18 critical factors for telemedicine service deployment [18], and its compliance state by the PCS. Assessment based on the collected evidence.

## Data Availability

The datasets used and/or analyzed during the current study are available from the corresponding author on request.

## Abbreviations

CHUC: Coimbra University Hospital Centre
CPH: Coimbra’s Pediatric Hospital
CPLP: Community of Portuguese-speaking countries
CVF: Competing Values Framework
ECG: Electrocardiography
Echo: Echocardiography
GP: General practitioners
ICT: Information and communication technology systems
NHS: Portuguese national healthcare services
OCAI: Organizational Culture Assessment Instrument survey
PALOP: Portuguese-speaking African countries
PCS: Pediatric Cardiology Service
SONHO: The main integrated data management information system in Portuguese hospitals
TC: Teleconsultation
UHC: Universal Health Coverage

## Footnotes

1 General practitioners (GP) in Portugal are actually specialty physicians in family medicine (4 years of specialization)

2 All participants provided verbal and written consent, as required. The CHUC’s board approved and welcomed the study

3 The S-shape logistic growth curve, associated with technology innovations, is defined by *P=K/(1+ exp-(a + bt)),* where *P* represents the cumulative performed TC, *k* the ceiling (empirically estimated until perfect fitting), *t* the time variable, a the constant integrating the curve on the time scale, and *b* the growth coefficient rate

4 SONHO, the main integrated data management information system in Portuguese hospitals. It was developed in the 1990s to support the administrative service of national public hospitals, allowing the export of several statistical indicators. Portaria 567/2006 introduced the legal context to telemedicine’s digital records in SONHO system

5 As the Pediatric Hospital’s Director explains in its speech for the hospitals’ 40th anniversary, June 1, 2017: “the first building built from root as a pediatric hospital in more than a century in Portugal”

6 The total avoidable travel cost, per round trip was estimated for the total of 16,249 TC, performed from 2007 to 2014.
