# Supplementary figures and images for: How to develop a sustainable telemedicine service? A Pediatric Telecardiology Service 20 years on - an exploratory study
Source: BMC Health Serv Res. 2019 Sep 23;19:681. doi: 10.1186/s12913-019-4511-5 (PMC6757431; doi:10.1186/s12913-019-4511-5)

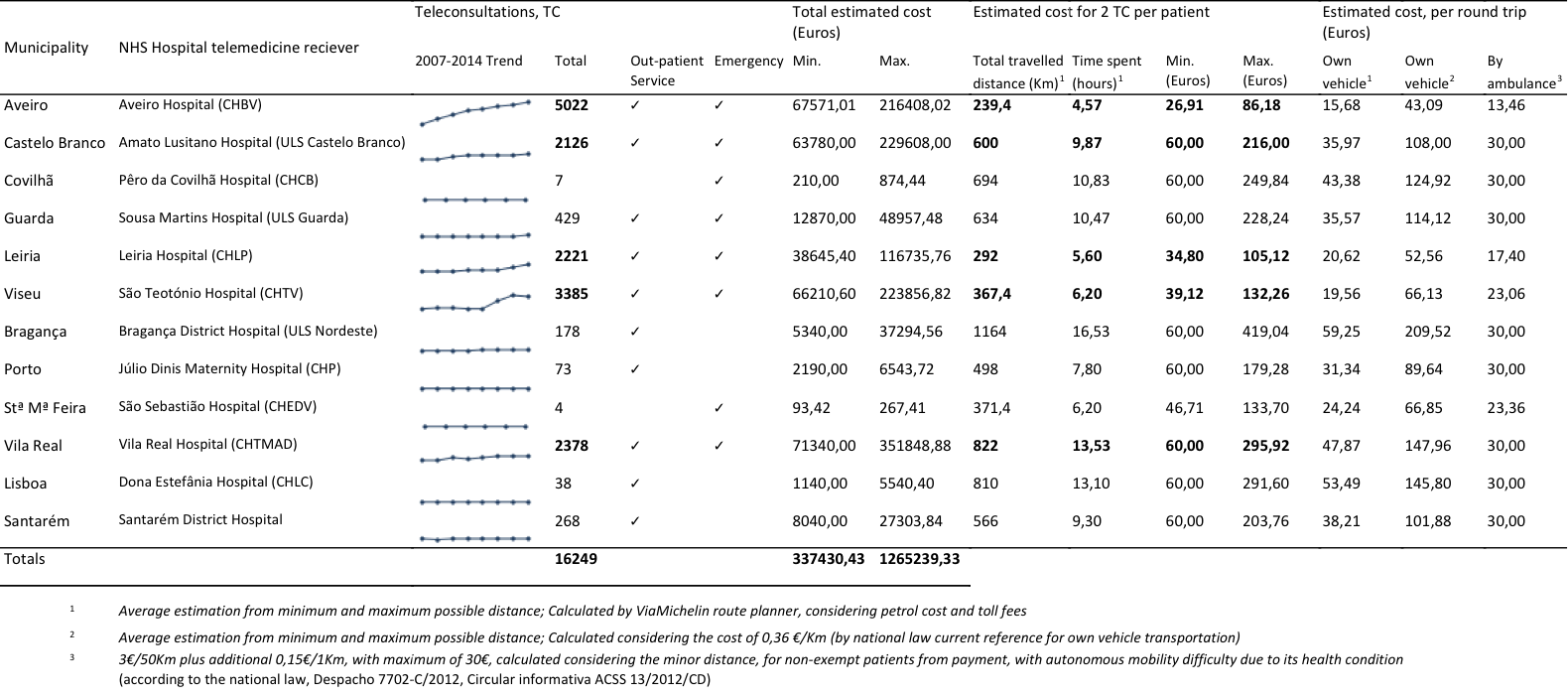

Supplement: Supplementary file 1 — Additional file 1. The cost impact of telemedicine for patients and their family, per municipality and totals. Represented through time spent, travelled distance and financial direct costs, calculated through three methods. Includes out-patient consultations and emergency acts, by telemedicine. [file 12913_2019_4511_MOESM1_ESM.png]

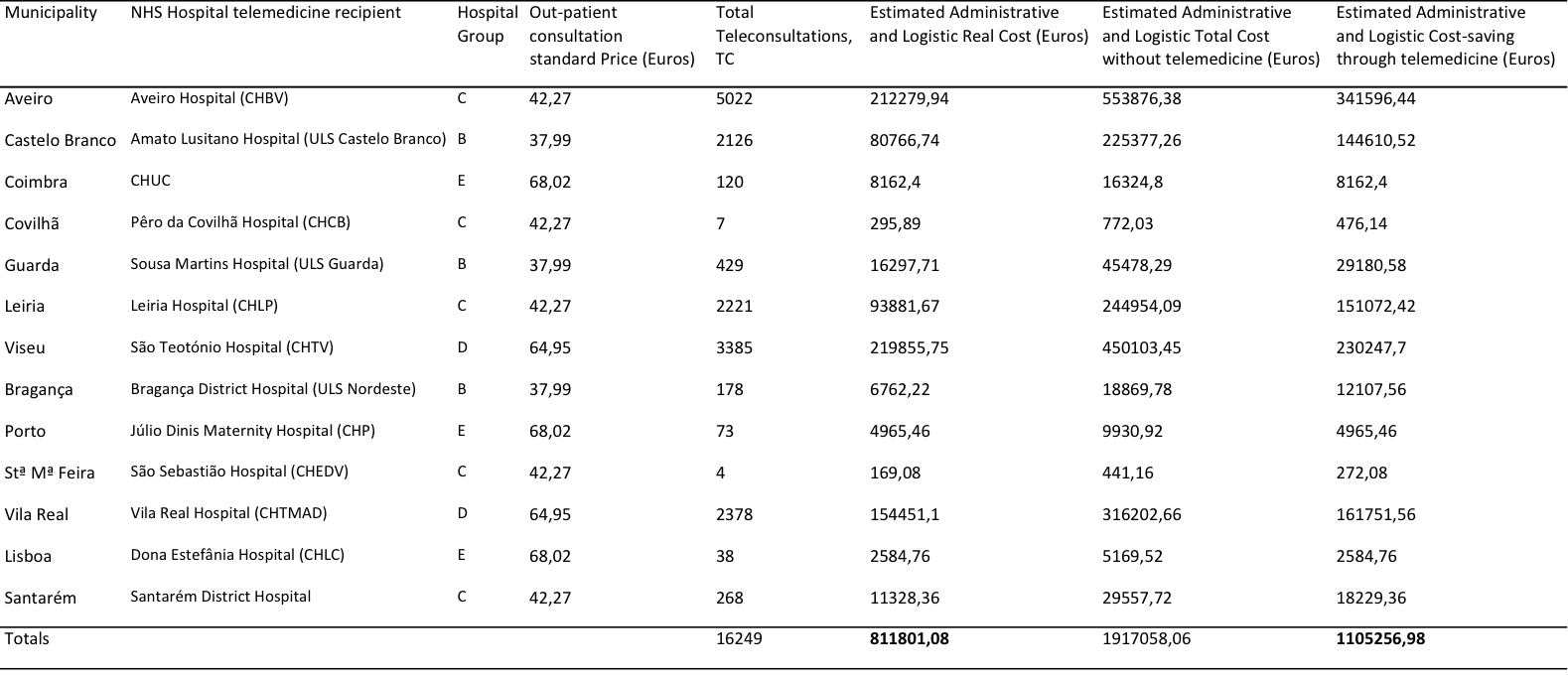

Supplement: Supplementary file 2 — Additional file 2. The organizational cost impact of telemedicine, for the system, per municipality and totals. Given by out-patient consultations’ price (representative of the clinical and administrative activities related with each consultation act), contracted by each hospital, according to the respective national norm [34]. Includes out-patient consultations and emergency acts, by telemedicine. [file 12913_2019_4511_MOESM2_ESM.png]

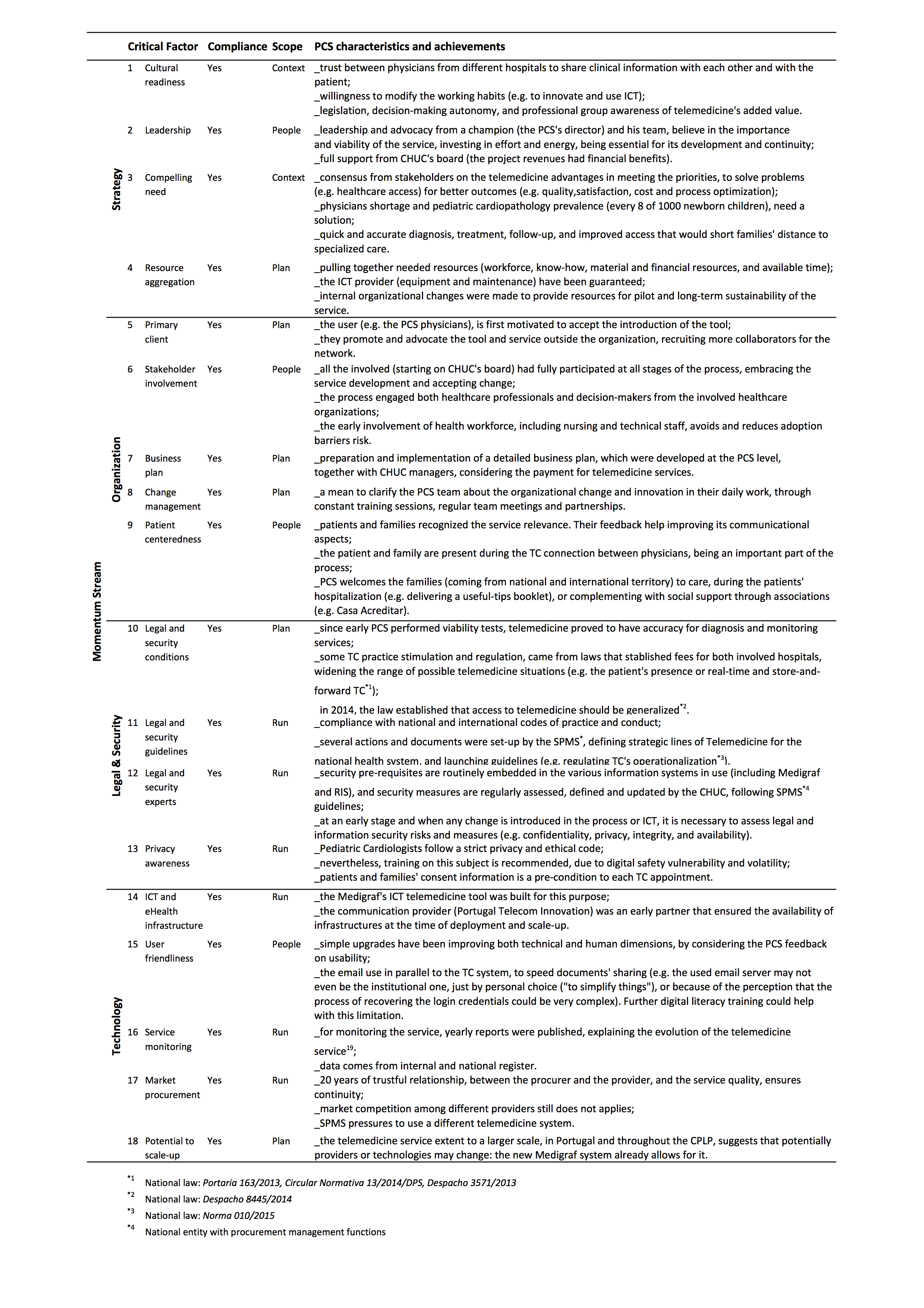

Supplement: Supplementary file 3 — Additional file 3. The Momentum’s 18 critical factors for telemedicine service deployment [18], and its compliance state by the PCS. Assessment based on the collected evidence. [file 12913_2019_4511_MOESM3_ESM.png]
